# ‘What is Inconvenient for You is Life-saving for Me’: How Health Inequities are playing out during the COVID-19 Pandemic

**DOI:** 10.1007/s41649-020-00119-1

**Published:** 2020-05-16

**Authors:** Vicki Xafis

**Affiliations:** Centre for Biomedical Ethics, Yong Loo Lin School of Medicine, National University of Singapore, Singapore

**Keywords:** COVID-19, Health inequity, Social determinants of health, Social justice, Social disparities, Coronavirus pandemic, Structural injustice

## Abstract

The COVID-19 pandemic has had a significant impact globally. Most affected, however, are those individuals and groups routinely disadvantaged by the social injustice created by the misdistribution of power, money, and resources. Simple measures that prevent the spread of COVID-19, such as frequent hand washing and social distancing, are unavailable to millions of people in the wealthiest of nations and in the poorest of nations. Disadvantaged groups are impacted more directly and in disproportionately higher numbers due to existing poor health, and the disruption of services central to securing an income and an education will have lasting consequences for their futures. The unintended effect of exclusionary government policies is that privileged citizens and healthcare systems are also at greater risk. This paper seeks to highlight the impact of COVID-19 on those already suffering health inequities through consideration of some of the social determinants of health on groups in affluent and poorer nations. It also highlights some of the factors that may assist in tackling health inequities as we emerge from this pandemic.

## Introduction

Most if not all of the new public health challenges that we are facing – be it in the areas of communicable, maternal, perinatal and nutritional conditions, noncommunicable conditions or injuries – are directly related to how we organize our societies and live our lives, with inequities among and within populations again standing out. Inequities both fuel the emergence of new public health challenges and result from them. (Blas and Sivasankara Kurup 2010, 4)

The conditions in which people live out their lives from the womb to their death are collectively referred to as the social determinants of health and are socially and politically structured by the distribution of power, money, and resources (Marmot et al. 2008). Leading world experts acknowledge that the systematic misdistribution of power, money, and resources fuels social injustice causing millions of people worldwide to die prematurely; they also acknowledge that we are all morally obliged to urgently reduce the health inequities experienced by millions—from governments to international agencies, the business sector, and civil society (Marmot et al. 2008). The COVID-19 pandemic has also revealed that governments’ unwillingness to invest in closing the glaring social gaps created by unjust policies has had costly and avoidable consequences in terms of both human life and economies.

Health inequities are experienced in countries which lack adequate infrastructure including health and educational services, water and energy infrastructure, and financial infrastructure; they are, however, also manifestly evident in wealthy nations (OECD Health Policy Studies 2019). Certain sectors of the population are more likely to be affected by social policies and structures that persistently disable them from exiting the social circumstances, which lead to poorer health outcomes. Groups likely to experience poorer health outcomes due to exposure to multiple levels of disadvantage include, but are not limited to, the following: indigenous peoples; those born into poverty or on very low incomes; those living in rural and remote communities; those experiencing job and housing insecurity; people experiencing chronic mental illness, disabilities, or dependence on substances; prisoners; newly arrived migrants; refugees (NSW Department of Health 2004) as well as displaced populations, stateless people, and migrant workers. Even if not driven to address health inequities by a sense of justice and moral obligation, one only needs to consider the economic costs arising from life-long treatments of chronic disease attributable in large part to social determinants of health (Woolfenden et al. 2013) to take action.

Worldwide, millions have been ‘inconvenienced’ by mandatory stay-at-home orders/lockdown measures/circuit breaker measures with some in the USA now rebelling against their civil rights being violated (Shubber and Sevastopulo 2020). For millions of people around the world, however, a safe space in which they could abide by the WHO guidance would prove lifesaving:

Wash your hands frequently; Maintain social distancing; If you have fever, cough and difficulty breathing, seek medical care early; Stay informed and follow advice given by your healthcare provider. (World Health Organization 2020)

For example, a displaced person living in an over-crowded refugee camp with a lack of basic infrastructure for sanitation and hygiene cannot avoid infection in the case of a local outbreak; he cannot wash his hands frequently, he cannot maintain social distancing, nor can he ring a health provider for advice, much less seek medical attention. He relies on nothing more than luck to survive.

This pandemic has exposed how reliant we all are on each other, how the health of the disadvantaged impacts on the advantaged, how events in one country impact on lives in others, how economies are impacted by the health of the people whose labour they rely on and on the health of those excluded from the labour market, and how we can only fight some battles standing united. With this renewed knowledge and insight, we can perhaps now address long-standing health inequities. Decisively, addressing the social determinants of health will involve local and international contributions, as health inequities exist locally, nationally, regionally, and globally. All sectors of society will need to play their part: from all areas of government (and not just health departments), the business sector, civil society, and local communities, as well as international agencies, such as the World Health Organization (Marmot et al. 2008).

This paper explores ways in which certain groups who already experience disadvantage are being affected by COVID-19 through a brief examination of some of the social determinants’ health that impact health outcomes in both affluent and poorer nations. The areas considered include the following: income and wealth, employment and access to health services, housing, food environment, education, and safety. Examples are given to highlight the way health inequities play out for different groups but it should be remembered throughout that one form of disadvantage often leads to being impacted by other forms. For example, inability to access education will most often impact on health, and life more broadly, in numerous direct and indirect ways at various stages of a person’s life.

## The Impact of Health Inequities during the COVID-19 Pandemic

Health inequities experienced in childhood persist into adulthood producing poor physical and mental health outcomes. For example, children that have experienced poverty are more likely to suffer higher mortality rates (particularly if poverty persists in adulthood), a greater incidence of cardiovascular disease, of stroke, and type II diabetes (Raphael 2011). Exposure to other forms of childhood adverse experiences, such as family dysfunction and cohabiting with individuals who suffer mental illness or who abuse substances, also takes their toll on the health and well-being of a child right into adulthood (Loxton et al. 2019; Bellis et al. 2017). In the face of a pandemic such as COVID-19, groups systematically disadvantaged confront the virus with a health status already compromised compared with less disadvantaged groups (Bailey et al. 2017). For example, Hispanic Americans and African Americans have succumbed to COVID-19 in disproportionately higher numbers (New York State Department of Health 2020; Yancy 2020) than their fellow Americans; at the time of writing, 34% of fatalities in New York City were Hispanics (29% of the total population) and 28% of fatalities were African Americans (22% of the total population) while 27% of fatalities were White (32% of the total population) (New York State Department of Health 2020, 19 April 2020).

Also at significant risk during a pandemic are indigenous peoples the world over, as their health is significantly impacted in numerous profound ways; life expectancy is lower, infant and child mortality is high, as is maternal morbidity and mortality; there are high levels of chronic disease, increased levels of substance abuse, malnutrition, high levels of depression, and susceptibility to infections (Valeggia and Snodgrass 2015). Exceptional measures have been taken in some countries (e.g. Australia) such as closing off communities and banning all travel unless it falls under limited restricted categories to ensure that indigenous communities are protected from COVID-19 (National Indigenous Australians Agency 2020; Department of Health, Australian Government 2020). Like other indigenous peoples, for example, communities of the APY Lands in South Australia are also at increased risk. The risk, however, is further amplified because the elders in these communities are also community leaders who hold much of the history and culture; hence, losing them would cause irreversible devastation to these communities (ABC News 2020). In contrast to the strategy adopted in Australia, the Brazilian Government has done nothing to protect numerous indigenous communities in the Amazon, many of which are now at grave risk of being decimated by the virus (Wallace 2020). In fact, the Bolsonaro Government has turned a blind eye to illegal practices such as mining and logging (Wallace 2020), practices which imperil these vulnerable indigenous communities (and the whole world) both during a pandemic and beyond such a crisis.

The following sections briefly focus on some social determinants of health which, on their own and outside a pandemic, impact significantly and across time and generations on the physical and mental health and well-being of individuals and groups. In the presence of a pandemic, however, the effects are magnified and the consequences are more devastating.

### Income and Wealth

Poverty is linked with poor health outcomes, which are often intergenerational (OECD Health Policy Studies 2019). Individuals experiencing entrenched poverty often rely on welfare as their only source of income but welfare policies in wealthy nations discourage welfare reliance by providing payments that do not adequately cover even the most basic of needs. This stance was abundantly expressed in a recent Australian government-funded report (Wilkins et al. 2019): ‘It [i.e. reliance on welfare] is associated with significant demands on government budgets and reduced economy-wide market output’.

COVID-19 has presented governments with significant conflicting problems: in order to prevent crippling the healthcare systems, it was necessary to take unprecedented isolation measures which have had a devastating impact on the economy as unemployment soared (Aaronson and Alba 2020) and queues at unemployment offices resembled those of the Great Depression (Wright and Bagshaw 2020). Governments responded decisively offering a range of relief measures including unemployment benefits, which in some jurisdictions doubled overnight (Lawson 2020; Services Australia 2020) despite years of resistance to do so even when urged to by organisations such as The Australian Council of Social Service and the Business Council of Australia (Business Council of Australia 2012; Murphy 2020).

Critically important measures should rightly be adopted to prevent the collapse of economies. However, the ease with which billions, and even trillions, of dollars were sourced overnight prompts some reflection on governments’ long-standing resistance to tackling health inequities decisively, especially given the uncontested link between better health and greater productivity (OECD Health Policy Studies 2019).

### Employment and Access to Health Services

Millions of people have been confronted by sudden, unexpected unemployment and are heavily relying on governments to provide some assistance, as noted above. When individuals of a low socio-economic status do secure employment, it is often manual labour, which precludes them from working from home, a protection measure for themselves, their families, and the broader community during this crisis.

The COVID-19 pandemic has either resulted in the loss of jobs, devastating poor families reliant on the income, or has forced workers to continue working at the frontline in low-paid cleaning, delivery, transport, supermarket/grocery jobs, or factory labour, often exposing workers to increased risks of contracting the disease (Lussenhop 2020; Ducharme 2020; Channel News Asia 2020). In addition to increased exposure to the virus, these workers are also at higher risk because they are often unable to access free healthcare and paid sick leave, which could encourage them to continue working even if unwell (Parker 2020). Even where jobs have survived, single-parent families with young children are potentially still at risk of losing their jobs, as social distancing measures preclude family members from looking after young children who cannot be left at home unattended. While holding a job during this crisis is life-saving for poorer families, the very fact that the bread winner is more exposed to contracting the disease also exposes whole families to this risk, which is particularly high when large or extended families occupy sub-optimal housing, as discussed below.

In low- to middle-income countries (LMIC), COVID-19 has caused sectors employing thousands, such as the textile industry and the tourism industry in Sri Lanka, for example, to grind to a standstill due to the drop in demand for products and travel restrictions crippling the tourist industry (PWC 2020). As is the case in other LMICs, linked to and completely reliant on these industries are the self-employed whose daily earnings support whole families, for example, the food stall owner, the souvenir stall owner, and the tuk driver, none of whom would be considered small and medium enterprises eligible for government assistant packages (PWC 2020).

### Housing

The physical infrastructure we call ‘housing’ takes many forms to include independent structures in suburbs or cities, temporary settlements accommodating refugees or displaced peoples, and shelters in slums (World Health Organization 2018) or migrant dormitories such as those in Singapore (Chew 2020). When we consider the risks specific to housing, it is important to remember that the occupants of these dwellings are deprived of other life-saving aspects many of us take for granted, such as safety and security, an income, and food.

People with limited financial means typically struggle to gain access to safe and affordable housing and, in wealthy countries, often rely on social housing programmes subsidised by the government (Department of Social Services 2020). Housing offers enormous practical benefits from sheltering from the elements, enabling the storage and cooking of meals, providing water and sanitation (for some) (Jacobs 2011), to existential benefits, as housing shields us from the gazing eyes of the world ensuring that we experience the privacy we need to flourish as humans (Reiman 1976). Many of these forms of housing lack the very basic features that promote health and prevent the spread of infectious diseases: water, sanitation, and hygiene (World Health Organization 2018).

A consistent feature of underprivileged people’s living arrangements is overcrowding, which has enormous physical and mental health impacts on occupants of overcrowded dwellings (World Health Organization 2018). The spread of COVID-19 is accelerated in overcrowded dwellings, settlements, shelters, and dormitories where it is impossible to practice social distancing measures. Millions of people around the world are at imminent risk should the virus find its way into refugee camps, slums, and temporary settlements both because of overcrowding and particularly because water and sanitation infrastructure is lacking, particularly worrying with evidence that the virus is also transmitted via faeces (Chen et al. 2020).

The most extreme exclusion from any form of housing (and involvement in society) is homelessness, whose definition in many countries is broader than simply *living in public spaces* to also include temporary living arrangements in shelters or emergency accommodation (OECD 2020). Homeless people often have no secure access to water, sanitation, or hygiene and are at great risk. It has become evident that crowded homeless shelters, while providing some protections under normal circumstances, cannot help protect against COVID-19. This has prompted some cities to move homeless people to hotels to help prevent the spread of the virus (Siebert 2020).

Added to these significant risks is the inability that millions of people with insecure housing have to access healthcare both in wealthier and poorer nations. Despite the efforts of humanitarian organisations, such as Médecins Sans Frontières (2020) and the United Nations (UN Department of Global Communications 2020), millions around the world will not benefit from medical attention.

### Food Environment

The link between food consumption and health may seem straightforward. However, there are different levels of inadequate food consumption. The general population of many developed countries does not consume a sufficient variety of foods from the various food groups recommended by health authorities, often leading to the development of chronic conditions (Australian Institute of Health and Welfare 2018). For millions of people around the world, however, the concern is of a more urgent nature; there is simply not enough food. According to the World Food Programme, a staggering 821 million people are undernourished, i.e. ‘…an individual’s habitual food consumption is insufficient to provide the amount of dietary energy required to maintain a normal, active, healthy life’ (FAO, IFAD, UNICEF, WFP, and WHO 2019, 189). These people do not have the ability to secure access to food and hence suffer from what is widely known as ‘food insecurity’, which is also experienced by millions of people in prosperous countries, for example, by low income earners who are often of specific ethnic backgrounds and have a lower education (Gundersen and Ziliak 2015), and unsurprisingly, homeless people (Crawford et al. 2014). In addition to creating a new category of people in need of emergency food because of job losses, this pandemic has exacerbated food insecurity experienced by disadvantaged groups in wealthier nations but also by those depending on international aid as a result of supply chain disruptions and movement restrictions.

Children are particularly vulnerable, especially during a pandemic. School closures due to the COVID-19 pandemic will impact the lives of millions of children many of whom are already nutritionally disadvantaged and who heavily rely on school food programmes (WFP, FAO, and UNICEF 2020). Important joint guidance to governments has been issued by the World Food Programme, the Food and Agriculture Organisation of the UN, and by UNICEF (WFP, FAO, and UNICEF 2020) to ensure the safety and health of children during the pandemic.

Also, particularly at risk are women who are the sole bread winners for their families. They are potentially more likely to be affected by the multiple impacts of the COVID-19 pandemic, as food prices rise to levels they cannot afford, as social distancing measures impact on their ability to fetch food and water, and as humanitarian food supplies may be disrupted by border closures or the lack of funds to swiftly implement emergency control measures (Nevill 2020).

### Education

More than 850 million school and university students are not attending classes due to COVID-19, which UNESCO estimates roughly half the worldwide student population (UN Department of Global Communications 2020). According to another source, however, this figure is 1.5 billion children with 180 countries enforcing nationwide school closures (WFP, FAO, and UNICEF 2020). Education plays a particularly significant role in children and adolescents’ health and well-being and has a lasting impact on their health into adulthood, even if the mechanisms and mediating pathways are still not fully understood (Hamad et al. 2018).

Disadvantaged children and young people have fewer chances of receiving a good education due to a web of socio-economic factors, which impact on their ability to benefit from schooling and to remain in school. For example, the adverse impact of social factors such as poverty is significant, as it affects child development as well as children and adolescents’ cognitive development and increases the risk of self-harm as well as engagement in violent criminal behaviours (Najman et al. 2009; Mok et al. 2018). However, receiving an education can help curb such influences. For example, it has been suggested that cognitive functions such as reasoning, thinking, and problem-solving which education by its very nature entails provide a direct protective benefit, while indirect benefits accrue from educated students later having access to better jobs, more control over their lives, and a greater ability to make healthier life choices (Ross and Wu 1995).

For millions of students around the world, the COVID-19 pandemic has necessitated the adoption of remote schooling and distance learning. However, the most disadvantaged children and young people are not able to participate in such learning simply because they either have no access to the internet or do not have the ability to purchase the hardware required. Even where internet access and harware are not an issue, remote learning requires parental involvement and support, which is often not available in some families due to complex socioeconomic factors; it requires adequate dedicated space for study, an environment free of disruption, and multiple devices for families with more than one child. The absence of such features is sure to have a negative long-term impact on children already disadvantaged academically and socially with a subsequent impact on their health and well-being, especially if remote schooling extends over a long period. Children whose countries have not instituted closures may also be impacted adversely, as their parents may be reluctant to send them to school for fear of them contracting the disease (WFP, FAO, and UNICEF 2020).

The disruption to these children’s lives is more significant than simply not having structure to their day for the duration of the stay-at-home orders/lockdown; for some children and young people whose school attendance is irregular at the best of times, the disruption caused by COVID-19 could lead to them abandoning school altogether resulting in significant life-long health and wellbeing consequences.

### Safety

Domestic and family violence as well as child abuse and neglect are associated with social determinants of health, such as significant financial strain, and secondary consequences of social determinants of health, such as mental illness and drug and alcohol abuse (State of Victoria 2016). Both men and women experience domestic violence but women tend to be victims more frequently (State of Victoria 2016).

The requirement to socially isolate and constantly remain in the same space as one’s abuser has inevitably placed many women and children at increased risk of harm from domestic and family violence with serious long-lasting consequences for victims’ physical and mental health (Abramson 2020). Domestic and family violence come in many forms, and during the COVID-19 pandemic, life has become increasingly more dangerous, as victims cannot escape the attention of their abuser, cannot take refuge at other family members’ homes, may have fewer funds due to job losses, and may have greater difficulty contacting helplines despite efforts made by domestic violence services to provide a range of contact modes (Godin 2020). Some measures have been taken to help address such violence. For example, as in other countries, in the EU, there have been urgent calls to increase emergency accommodation for women at risk, to support member states financially to achieve this, and to assist with appropriate communication of the supports available (Women’s Rights and Gender Equality 2020).

## Deep Insight and a New Opportunity

This pandemic has already brought about a new awareness. For example, it is only now that low paid workers have finally been recognised for the vital contribution they make to keeping cities and countries functional. People we have previously walked past as if they were invisible have suddenly come into sharp focus; the supermarket worker stacking the shelves has become our hero because he not only replenishes stocks but, in doing so, also unwittingly provides psychological relief; the homeless person lying in the entrance of a building has suddenly become the subject of our concern…or of our fear; the factory worker who works tirelessly to make the mask which may save our life is another such hero.

The COVID-19 pandemic has demonstrated, in profound ways, that all sectors of society and all members of society are interlinked and interdependent. The risks disadvantaged members of societies are exposed to have impacted the whole of society with devastating consequences on mortality and on healthcare systems which, in many parts of the world, have struggled to cope with the rapid rates of infection. Despite the global turmoil COVID-19 has created, governments around the world are now presented with an opportunity to correct the health inequity course we have been on for decades.

## Concluding Remarks

In the months ahead, governments will need to reflect on what has become the greatest challenge of our lifetime. Had governments of wealthy nations invested with commitment in programmes and policies that aim to reduce health inequity, thousands of lives may have been spared and billions of dollars may have been saved. The reduction of health inequities is an ethical imperative but is also prudent from an economic sense, as it ultimately leads to greater productivity, less strain on healthcare systems, and fewer welfare programmes.

It will never be possible to fully eradicate health inequity. However, the multidisciplinary approach that needs to be taken from the start of a child’s life has been articulated multiple times for multiple groups by world renowned experts and focuses on ‘early child development; education and skills development; employment and working conditions; minimum income for healthy living; sustainable communities; and a social-determinants approach to prevention.’ (Marmot 2011). Closing the health inequity gap will entail many years of dedicated efforts but governments around the world now have a rare opportunity to achieve this.

Finally, it has emerged that the character traits of our leaders have played a decisive role in responding to this pandemic. Likewise, during the recovery efforts, leaders around the world will either guide us prudently through this lengthy phase or contribute to widening the health inequity gaps locally and worldwide.
